# Evaluation of long-term supplementation of a *Bacillus subtilis* direct-fed microbial and enzymatically hydrolyzed yeast cell culture product used alone or in combination on *Clostridia*, *Clostridium perfringens*, *Escherichia coli*, and *Salmonella* prevalence in beef steers

**DOI:** 10.1093/jas/skae156

**Published:** 2024-06-03

**Authors:** Erin R DeHaan, Jesse Thompson, Warren C Rusche, Mackenzie de Jesus, Elliot Block, Tom Rehberger, Zachary K Smith

**Affiliations:** Department of Animal Science, South Dakota State University, Brookings, SD 57007, USA; Arm & Hammer Animal Nutrition, Church and Dwight Company, Princeton, NJ 08540, USA; Department of Animal Science, South Dakota State University, Brookings, SD 57007, USA; Arm & Hammer Animal Nutrition, Church and Dwight Company, Princeton, NJ 08540, USA; Arm & Hammer Animal Nutrition, Church and Dwight Company, Princeton, NJ 08540, USA; Arm & Hammer Animal Nutrition, Church and Dwight Company, Princeton, NJ 08540, USA; Department of Animal Science, South Dakota State University, Brookings, SD 57007, USA

**Keywords:** beef, *Clostridia*, direct-fed microbial, *E. coli*, enzymatically hydrolyzed yeast cell culture product, *Salmonella*

## Abstract

The objective was to determine the influence of long-term supplementation (258 d) of a direct-fed microbial (**DFM**) and/or yeast cell wall (**YCW**) product on bacterial populations in beef steers. Single-sourced Charolais × Red Angus steers (*n* = 256; body weight = 246 ± 1.68 kg) were used in a randomized complete block design and blocked by location into one of four treatments: 1) fed no DFM and no YCW (Control); 2) fed only the DFM (DFM; Certillus CP B1801 Dry, 28 g/steer d^−1^ ); 3) fed only the YCW (YCW; Celmanax; 18 g/steer d^−1^ ); and 4) fed the DFM and the YCW (DFM+YCW). Steers were vaccinated for respiratory and clostridial diseases and treated for internal and external parasites at processing and individually weighed on days 1, 14, 42, 77, 105, 133, 161, 182, 230, and 258. To determine bacterial prevalence, fecal samples were collected on days 1, 14, 77, 133, 182, and 230 and environmental (pen area, feed, and water) samples were collected at the beginning of the week when cattle were weighed. No treatment × day interactions or treatment effects (*P* > 0.05) were observed between treatment groups at any sampling days for the bacterial populations. Samples on days 1, 133, and 182 had greater (*P* < 0.05) *Clostridia* levels compared to the other sampling points but were not different from each other. *Clostridia* levels were also greater (*P* < 0.05) on day 77 compared to days 14 and 230. Samples on days 77 and 230 had greater (*P* < 0.05) *Clostridium perfringens* levels compared to the other sampling points but were not different (*P* > 0.05) from each other. Samples on days 1 and 14 had lower (*P* < 0.05) total *Escherichia coli* levels compared to the other sampling points but were not different (*P* > 0.05) from each other. *Escherichia coli* levels on day 77 were higher (*P* < 0.05) compared to days 133, 182, and 230. Little *Salmonella* prevalence (1.5%) was observed throughout the study. This study had greater levels of *Clostridia* compared to small and large commercial feedlots in the Church and Dwight research database, but *C. perfringens*, total and pathogenic *E. coli*, and *Salmonella* prevalence were notably lower. Collectively, there were no appreciable treatment influences on bacterial populations. These data further indicate a low pathogenic bacterial challenge at the trial site, which could partially explain the lack of differences with DFM or YCW supplementation. The DFM and YCW used alone or in combination cannot be expected to show additional benefits when animals are relatively unstressed with a low pathogenic bacterial challenge.

## Teaser

The DFM and YCW used alone or in combination in feedlot beef steers did not show productive benefits when animals were relatively unstressed with a low pathogenic bacterial challenge.

## Introduction

Foodborne pathogens have accounted for approximately 9.4 million illnesses annually with 55,961 cases that resulted in hospitalizations and 1,351 of the illnesses ended in death (Scallan et al., 2011). Of the illnesses reported, 11% were attributed to *Salmonella* and 10% were to *Clostridium perfringens*, as well as 35% of hospitalizations were attributed to *Salmonella*. *Salmonella* was also the leading cause of foodborne pathogen-related deaths accounting for approximately 28% of deaths (Scallan et al., 2011). Pathogenic bacteria (i.e., *Salmonella*, *Escherichia coli*, and *C. perfringens*) are known to reside in the gastrointestinal tract of cattle that appear healthy (i.e., asymptomatic). Feces and carcass-associated lymphatic tissue are potential sources of food supply contamination as *Salmonella* in particular can reside in lymph nodes (Xie et al., 2016). In January 2020, a citizen petition requested the USDA Food Safety and Inspection Service to declare 31 *Salmonella* strains as adulterants in meat products (FDA, 2022). Should this come into action, this could pose problems for cattle producers as 4.8% of foodborne *Salmonella* outbreaks can be attributed to beef products (Xie et al., 2016). Antimicrobials have been used in livestock feeds in an attempt to combat this issue. Antimicrobial resistance is a great concern to animal producers, livestock product processors, and consumers. Continued and unwarranted use of antimicrobials in livestock production results in increased pools of antimicrobial-resistant genes among bacteria. Specifically, antimicrobial resistance in pathogenic bacteria capable of causing foodborne illness is of greatest concern to beef producers, processors, and consumers. As *Salmonella*, *E. coli*, and *C. perfringens* are common pathogens found in cattle feces and are capable of causing foodborne illness (Flach et al., 2022), it is essential to find alternative preharvest interventions to reduce these pathogen loads and help improve food safety. *Bacillus subtilis*-based direct-fed microbial (**DFM**) feed additives have been shown to reduce harmful pathogenic bacteria in the gastrointestinal tract (Smock et al., 2020; Smith et al., 2021). Yeast cell wall (**YCW**) components of *Saccharomyces cerevisiae* have been shown to reduce inflammation and modulate immune function while also helping reduce pathogen loads (Baines and Erb, 2013; Stefenoni et al., 2020). While preharvest interventions are not expected to completely eliminate pathogens, there is potential to reduce pathogen populations below threshold capacity (Arthur et al., 2010). There is potential that the use of these products alone or in combination can aid in controlling systemic inflammation that in turn might reduce the need to use in-feed antimicrobials to control disease. The objective of this research was to determine the influence that long-term supplementation of a DFM and YCW fed alone or in combination have on bacterial populations in beef steers fed in confinement from weaning to harvest.

## Materials and Methods

### Institutional animal care and use approval

This study was conducted at the Ruminant Nutrition Center (**RNC**) in Brookings, SD, USA. The animal care and handling procedures used in this study were approved by the South Dakota State University (SDSU) Animal Care and Use Committee (Approval Number: 2009-044A).

### Cattle management

Single-sourced, newly weaned Charolais × Red Angus steers (*n* = 256) from Western South Dakota were transported approximately 513 km to the RNC in Brookings, SD in October 2020. This study used eight replicate pens per treatment and each pen contained eight steers (*n* = 64 steers per treatment). Steers were used in a randomized complete block design and blocked by location into one of four treatments: 1) fed no DFM and no YCW (Control); 2) fed only the DFM (DFM; Certillus CP B1801 Dry, *Bacillus subtilis* and *Lactobacillus plantarum* based DFM; 28 g/steer d^−1^ ); 3) fed only the YCW (YCW; Celmanax, enzymatically hydrolyzed yeast component of *Saccharomyces cerevisiae*; 18 g/steer d^−1^ ); and 4) fed the DFM and the YCW (DFM+YCW; Certillus 28 g/steer d^−1^ and Celmanax 18 g/steer d^−1^ of Celmanax). Supplements for dietary treatment inclusion were manufactured every 30 d at the SDSU feed mill in Brookings, SD. Upon manufacturing of the supplement, samples (Type B additive) were sent to Arm & Hammer Animal Nutrition in Wauwatosa, WI for confirmatory testing of Certillus. Diets fed to steers throughout the study are listed in Table 1. For additional information about cattle management, dietary treatments, growth performance, and carcass trait determination, please refer to Gubbels et al., (2023).

**Table 1.** Diet formulation of diets offered to steers during each period of the study^1^

Item	Days 1 to 42 (receiving)	Days 43 to 112 (growing)	Days 113 to 119 (transition 1)	Days 120 to 126 (transition 2)	Days 127 to 230 (finisher 1)	Days 231 to 245 (finisher 2)	Days 246 to 258 (finisher 3)
Dry-rolled corn, %	—	14.68	16.85	24.33	32.74	35.49	69.10
High moisture corn, %	—	—	15.65	22.56	32.43	34.98	—
Dried distillers grains with solubles, %	19.21	19.70	17.19	15.13	15.03	15.29	15.11
Corn silage, %	52.15	51.65	42.61	30.34	12.25	—	—
Oat hay, %	18.96	4.75	—	—	—	—	1.99
Sorghum silage, %	—	—	—	—	—	6.80	6.46
Pelleted supplement^2^, %	5.82	6.54	—	—	—	—	—
Liquid supplement^3^, %	—	—	5.14	5.10	5.24	5.27	5.24
Treatment supplement^4^, %	3.87	2.70	2.57	2.55	2.32	2.12	2.10
Diet composition^5^							
DM, %	47.81	50.91	49.25	56.02	68.24	71.52	77.90
CP, %	12.66	13.26	13.72	13.31	13.21	13.14	13.12
NDF, %	43.31	37.94	29.86	25.07	18.48	17.12	18.09
ADF, %	26.81	19.59	14.11	11.63	11.17	9.28	9.75
Ash, %	6.56	6.19	6.26	5.75	5.35	5.36	5.44
EE, %	2.61	2.48	3.55	3.49	3.28	3.40	3.42
NEm, Mcal/kg	1.73	1.86	1.93	2.01	2.11	2.12	2.07
NEg, Mcal/kg	1.08	1.21	1.29	1.36	1.44	1.45	1.40

^1^Each treatment was offered the same diet with the exception to the treatment supplement and ractopamine hydrochloride was fed at a rate of 300 mg/steer daily during the final 28 d on feed (day 230 of the study).

^2^Pelleted supplement contained exclusively soybean hulls.

^3^Liquid supplement (all values except for DM on a DM basis): 69.04% DM, 41.86% crude protein, 38.38% nonprotein nitrogen, 0.95 Mcal/kg of NE for maintenance, 0.66 Mcal/kg of NE for gain, 10.89% calcium, 0.32% phosphorus, 7.00% potassium, 0.22% magnesium, 6.03% NaCl, 0.33% sulfur, 4.23 ppm cobalt, 199.88 ppm inorganic copper, 11.99 ppm iodine, 15.07 mg/kg EDDI, 83.16 ppm iron, 304.81 ppm manganese, 2.90 ppm selenium, 664.59 ppm inorganic zinc, 44,064.55 IU/kg vitamin A, 376.52 IU/kg vitamin E, and 638.6 g/Mg monensin sodium.

^4^Soybean hull carrier supplement with either 0 g/steer (CON), Certillus at 28 g/steer d^–1^ (DFM), Celmanax at 18 g/steer d^−1^ (YCW), or Certillus at 28 g/steer d^−1^ and Celmanax at 18 g/steer d^−1^ (DFM + YCW).

^5^All values except for DM on a DM basis.

### Pathogenic prevalence

Fecal grab samples were collected on days 1, 14, 77, 133, 182, and 230 from three steers per pen that were closest to the pen mean body weight in order to evaluate *Clostridia*, *C. perfringens*, total *E. coli*, pathogenic *E. coli*, and *Salmonella* prevalence. Samples were collected from the same three steers per pen throughout the remainder of the study. Microbial environmental terroir samples were collected via bunk swabs, pipe and cable pen swabs, waterer swabs, pen floor grab samples, and feed ingredient samples. All environmental samples were taken on the first day of the week when the cattle were to be weighed and fecal grab samples collected (environmental samples were collected on Monday and cattle were weighed on Wednesday and Thursday). Swab samples were collected using a single sponge swab per collection site (bunk, pipe and cable, waterer, and pen floor) for each individual pen.

Samples were analyzed similar to McCarty et al., (2021) and are further described as follows. For *Clostridia*, samples were diluted 10-fold with sterile peptone and heat shocked for 35 min at 60 °C prior to enumeration on tryptose sulite cycloerine agar (Oxoid, Hampshite, UK) with d-cycloserine (400 mg/L) to select for *Clostridium* species. *Clostridia* counts were analyzed using 1:10 diluted samples and plates were anaerobically incubated at 37 °C for 24 h. Present isolated sulfite-reducing colonies were counted and up to five isolates per sample were harvested into reinforced clostridia medium (Oxoid, Hampshite, UK) and incubated anaerobically for 24 h at 37 °C. Following the 24 h incubation, cultures were transferred (10%) to brain heart infusion broth (BD, Heidelberg, Germany) and incubated anaerobically for 24 h at 37 °C. According to the manufacturer’s instructions, DNA was isolated as described by the Promega Maxwell high-throughput (**HT**) viral total nucleic acid (**TNA**) protocol (Maxwell HT Viral TNA kit; Promega, Madison, WI). The DNA was then screened for the alpha toxin gene specific to *C. perfringens* using polymerase chain reaction. Amplification of the toxin gene was analyzed using a previously validated PCR method (Yoo et al., 1997). The PCR mixture contained 2.5 µL 10× PCR Buffer, 2 µL of 50 mM MgCl2, 0.5 µL 10 mM dNTPs, 0.5 µM of each primer, 0.1 µL of Platinum Taq DNA Polymerase (Invitrogen, Carlsbad, CA), 2.5 µL of DNA, and sterile water was added to reach a total reaction volume of 25 µL. The mixture then underwent PCR at the following cycle settings: 5 min at 94 °C, 30 cycles of 94 °C for 1 min, 55 °C for 1 min, 72 °C for 1 min, and a final elongation of 3 min at 72 °C. Products from the PCR were observed using a Fragment Analyzer (Agilent Technologies, Santa Clara, CA) to determine the presence of the gene. An estimated *C. perfringens* count for each sample was calculated by dividing the total number of isolates positive for alpha toxin by the total number of representative isolates collected from that sample. This value was then multiplied by the total *Clostridia* count. *Clostridia* values below the 5 cfu/g limit were substituted with 1 cfu/g.

To determine *E. coli* enumeration, samples were 10-fold diluted with sterile peptone prior to enumeration on CHROMagar *E. coli* (CHROMagar, Paris, France) to select for *E. coli*, which grow as blue colonies. *Escherichia coli* counts were determined using 1:10 diluted samples, plates were aerobically incubated at 37 °C for 24 h. Present isolated *E. coli* were counted and up to five isolates per sample were harvested into tryptic soy broth (**TSB**; Heidelberg, Germany) and incubated aerobically for 24 h at 37 °C. Per manufacturer’s instructions, the DNA was isolated according to the Promega Maxwell HT viral TNA protocol (Maxwell HT Viral TNA kit; Promega, Madison, WI) and was then screened via two previously validated multiplex PCR methods for virulent genes (Johnson et al., 2008; Bai et al., 2012). Products from the PCR analysis were observed using a fragment analyzer (Agilent Technologies, Santa Clara, CA) to determine gene presence. An estimated pathogenic *E. coli* count for each sample was calculated by dividing the total number of virulent gene-positive isolates by the total number of representative isolates collected from that sample, and then that value was multiplied by the total *E. coli* count. *Escherichia coli* values below the method quantitation limit of 500 cfu/g were substituted with 100 cfu/g in the analysis.

For *Salmonella* testing, samples were enriched 1:10 with tetrathionate broth (Oxoid, Hampshite, UK) and incubated at 37 °C for 24 h prior to streaking on XLT-4 agar (Remel, Lenexa, KS). If black colonies were present, they were harvested as presumptive *Salmonella*, up to five isolates per sample were harvested into TSB (BD, Heidelberg, Germany) and incubated for 24 h at 37 °C. The inoculated TSB tube was then vortexed, the supernatant was pipetted into a new 15-mL conical tube, and centrifuged at 2,700 × *g* for 7 min. The supernatant was decanted and the pellet was resuspended in 200 µL 50 mM Tris-HCl (Sigma-Aldrich, St. Louis, MO) and 10 mM EDTA (Sigma-Aldrich, St. Louis, MO) solution. According to the manufacturer’s instructions, DNA was isolated as described by the Promega Maxwell HT viral TNA protocol (Maxwell HT Viral TNA kit; Promega, Madison, WI). DNA was then analyzed for the virulent gene *invA* via PCR to confirm each isolate was *Salmonella*. The PCR mixture contained 2.5 µL 10× PCR Buffer, 2 µL of 50mM MgCl2, 0.5 µL 10mM dNTPs, 0.3 µM of each primer, 0.1 µL of Platinum Taq DNA Polymerase (Invitrogen, Carlsbad, CA), 1 µL of DNA, and sterile water was added to attain a total reaction volume of 25 µL. The mixture then underwent PCR at the following cycle settings: 5 min at 94 °C, 35 cycles of 95 °C for 30 s, 60 °C for 30 s, 72 °C for 1 min, and a final elongation of 10 min at 72 °C. Products of PCR were observed using a fragment analyzer (Agilent Technologies, Santa Clara, CA) to determine the presence of the gene. If the *invA* gene was present for at least one of the isolates harvested from a sample, this was recorded as a *Salmonella*-positive sample. Conversely, if the *invA* gene was not present, the isolate was considered a false positive, and if no black colonies from a sample contained the *invA* gene, then it was recorded as a *Salmonella*-negative sample.

### Statistical analysis

All analyses were performed using Program R (R Core Team; Vienna, Austria, 2023) with the lme4, ggplot2, and emmeans packages. The effects of DFM and YCW on *Clostridia*, *C. perfringens*, total *E. coli*, pathogenic *E. coli*, and *Salmonella* prevalence were estimated using a mixed effects model with fixed effects of treatment, sample day, and their interaction; pen location as blocking factor; and pen was used for repeated measurements over time. Means were reported for each sample day averaging across treatment combinations and for each treatment combination within the sample day. *T*-tests were performed to compare averages using the Kenward-Roger method for degrees of freedom and *P*-values were adjusted for multiple comparisons using the Tukey method. Log odds ratios were computed to analyze concentrations of bacterial populations. Wald *z*-tests were performed to determine the significance of the log odds ratio and *P*-values were adjusted for multiple comparisons using the Tukey method. The total prevalence of bacterial populations were compared to large and small feedlots that have been previously sampled throughout trials by Church and Dwight and have been collected in a research database. An α level of 0.05 or less determined significance.

## Results and Discussion

Average bacterial counts (CFU/g) in fecal samples are listed in Table 2. There were no treatment × day interactions or treatment main effects (*P* > 0.05) observed for any of the analyzed bacterial populations. This suggests no beneficial or detrimental effects were observed with the use of either a bacillus-based DFM or YCW, alone or in combination.

**Table 2.** Average bacterial counts (CFU/g) in fecal samples collected from steers offered no DFM or YCW, only DFM, only YCW, or both DFM and YCW during a 258-d study^1^

Item	CON	YCW	DFM	DFM + YCW
*Clostridia* (CFU/g)				
Day 1	9,200	11,000	8,700	66,000
Day 14	4,300	6,200	3,700	3,000
Day 77	5,000	2,600	3,500	7,300
Day 133	9,100	7,200	11,000	5,600
Day 182	16,000	22,000	25,000	20,000
Day 230	800	350	520	24,000
*Clostridium perfringens* (CFU/g)				
Day 1	<10	100	<10	59,000
Day 14	8	<10	2	<10
Day 77	4,100	480	1,800	5,100
Day 133	400	120	3,800	1,200
Day 182	410	140	3,200	93
Day 230	740	290	390	24,000
Total *E. coli* (CFU/g)				
Day 1	550,000	375,000	220,000	990,000
Day 14	720,000	660,000	460,000	900,000
Day 77	4,100,000	4,400,000	38,000,000	7,100,000
Day 133	5,100,000	840,000	15,000,000	7,300,000
Day 182	800,000	2,400,000	1,400,000	2,200,000
Day 230	1,400,000	3,200,000	1,800,000	2,300,000
Pathogenic *E. coli* (CFU/g)				
Day 1	280,000	170,000	110,000	410,000
Day 14	310,000	110,000	250,000	370,000
Day 77	582,000	340,000	9,400,000	600,000
Day 133	3,300,000	67,000	370,000	2,900,000
Day 182	150,000	750,000	700,000	500,000
Day 230	7,200	440,000	290,000	290,000

^1^Treatments: fed no DFM and no YCW (CON); fed only the DFM (DFM; Certillus CP B1801 Dry, *Bacillus subtilis* and *Lactobacillus plantarum* based DFM; 28 g/steer d^−1^ ); fed only the YCW (YCW; Celmanax, enzymatically hydrolyzed yeast component of *Saccharomyces cerevisiae*; 18 g/steer d^−1^ ); and fed the DFM and the YCW (DFM+YCW; Certillus 28 g/steer d^−1^ and Celmanax 18 g/steer d^−1^ of Celmanax). Fecal grab samples were collected on days 1, 14, 77, 133, 182, and 230 of the study.

### Total *Clostridia*

**Figure 1.**
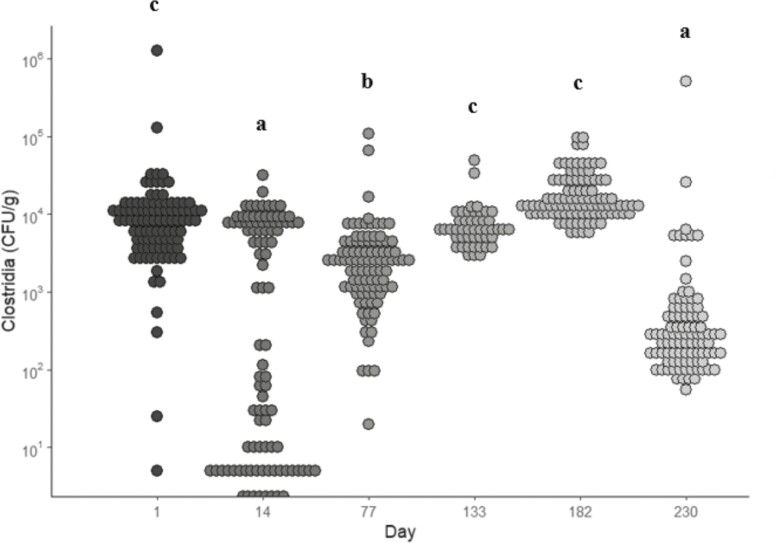
*Clostridia* prevalence (CFU/g) in fecal samples collected from all treatments during each sampling period. Treatments included: fed no DFM and no YCW (CON); fed only the DFM (DFM; Certillus CP B1801 Dry, *Bacillus subtilis* and *Lactobacillus plantarum* based DFM; 28 g/steer d^−1^ ); fed only the YCW (YCW; Celmanax, enzymatically hydrolyzed yeast component of *Saccharomyces cerevisiae*; 18 g/steer d^−1^ ); and fed the DFM and the YCW (DFM+YCW; Certillus 28 g/steer d^−1^ and Celmanax 18 g/steer d^−1^ of Celmanax). Fecal grab samples were collected on days 1, 14, 77, 133, 182, and 230 of the study. ^a,b,c^indicates day (*P* < 0.05).

Samples on days 1, 133, and 182 had greater (*P* < 0.05) *Clostridia* levels compared to the other sampling points but were not different from one another (Figure 1). *Clostridia* levels were also greater (*P* < 0.05) on day 77 compared to days 14 and 230. The sample from day 182 had the highest numerical level of *Clostridia* in the feed (corn silage) compared to the other sampling days (27,000 CFU/g vs. ≤110 CFU/g), however days 1 (20 CFU/g) and 133 (5 CFU/g) did not have a high *Clostridia* feed challenge detected. The high levels of *Clostridia* in the corn silage sample could provide a possible explanation for the high *Clostridia* levels observed in the fecal samples on day 182. However, this does not explain why levels were the highest on day 1 and remained at a high level on day 133. To our knowledge, this is the first study that has evaluated *Clostridia* levels specifically in feedlot cattle offered a *Bacillus*-based DFM and/or an enzymatically hydrolyzed yeast component of *S. cerevisiae*. Compared to previously sampled research sites, the fecal samples from the present study had a greater (*P* < 0.05) level of total *Clostridia* compared to small and large commercial feedlots in the Church and Dwight research database.

### Clostridium perfringens

**Figure 2.**
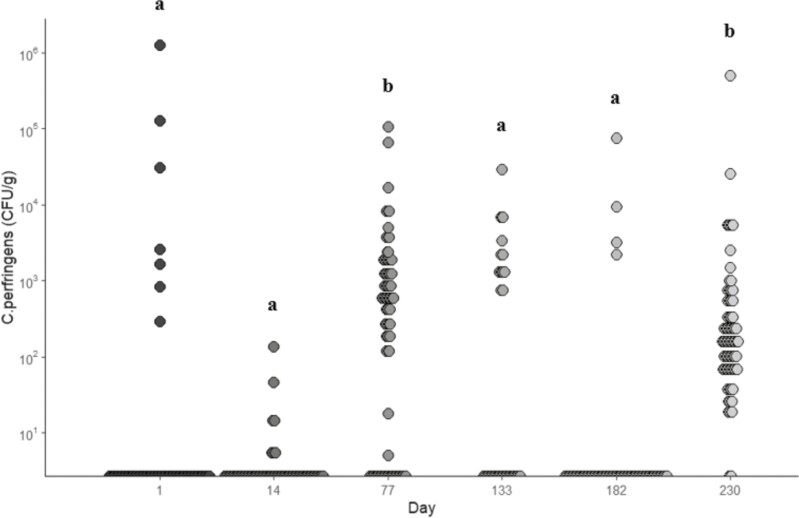
*Clostridium perfringens* prevalence (CFU/g) in fecal samples collected from all treatments during each sampling period. Treatments included: fed no DFM and no YCW (CON); fed only the DFM (DFM; Certillus CP B1801 Dry, *Bacillus subtilis* and *Lactobacillus plantarum* based DFM; 28 g/steer d^−1^ ); fed only the YCW (YCW; Celmanax, enzymatically hydrolyzed yeast component of *Saccharomyces cerevisiae*; 18 g/steer d^−1^ ); and fed the DFM and the YCW (DFM+YCW; Certillus 28 g/steer d^−1^ and Celmanax 18 g/steer d^−1^ of Celmanax). Fecal grab samples were collected on days 1, 14, 77, 133, 182, and 230 of the study. ^a,b^indicates day (*P* < 0.05).

Few studies have evaluated how *Bacillus subtilis*-based DFM’s and *S. cerevisiae* yeast culture products impact *C. perfringens* prevalence (Stefenoni et al., 2020; Flach et al., 2022). In the present study, treatment did not influence (*P* > 0.05) *C. perfringens* prevalence. However, Stefenoni et al. (2020) found that dairy cows supplemented with YCW had lower *C. perfringens* levels compared to control cattle that were not offered YCW. Samples in the present study on days 77 and 230 had greater (*P* < 0.05) *C. perfringens* levels compared to the other sampling points but were not different (*P* > 0.05) from each other (Figure 2). This was mainly dictated by prevalence as these two days had the most detectable *C. perfringens* in the fecal samples, while the other sample days had less *C. perfringens* detected in the fecal samples. Prevalence, or the percent of fecal samples that had detectable *C. perfringens*, was lower (*P* < 0.05) for this study (34.6%) compared to small (58.4%) and large (74.7%) commercial feedlots in the Church and Dwight research database. This is indicative of a lower pathogenic challenge during the trial.

Of the 2,451 *Clostridia* isolates from fecal samples collected throughout the trial, 556 were identified as *C. perfringens* (22.7%). The most abundant group of *Clostridium* throughout the trial was the *C. beijerinckii* group isolates with 1,308 isolates collected for a prevalence of 53.4%. However, this form of *clostridium* has been reported to be nonpathogenic and is considered to be a butanol-producer, which plays a role in producing enzymes for metabolic pathways (Patakova et al., 2019). *Clostridium perfringens* was the most abundant species in fecal samples on day 230 (70.0%). Overall, *C. perfringens* levels in feed and environmental samples were low (≤15,000 CFU/g) throughout the experiment. The low incidence of *C. perfringens* at this site and no differences observed between treatment groups could also provide further explanation for the lack of differences observed in health measures (Flach et al., 2022).

### Total and pathogenic *E. coli*

**Figure 3.**
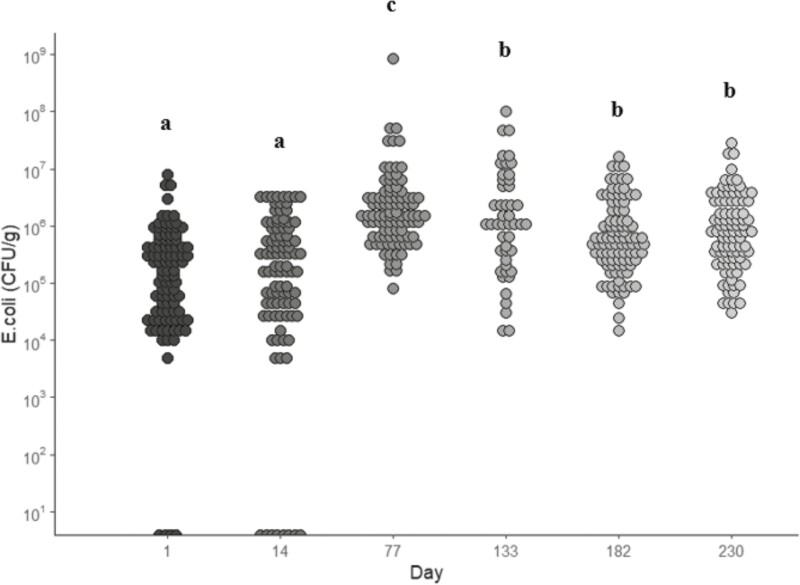
Combined *Escherichia coli* (*E.coli*) prevalence (CFU/g) in fecal samples collected from all treatments during each sampling period. Treatments included: fed no DFM and no YCW (CON); fed only the DFM (DFM; Certillus CP B1801 Dry, *Bacillus subtilis* and *Lactobacillus plantarum* based DFM; 28 g/steer d^−1^ ); fed only the YCW (YCW; Celmanax, enzymatically hydrolyzed yeast component of *Saccharomyces cerevisiae*; 18 g/steer d^−1^ ); and fed the DFM and the YCW (DFM+YCW; Certillus 28 g/steer d^−1^ and Celmanax 18 g/steer d^−1^ of Celmanax). Fecal grab samples were collected on days 1, 14, 77, 133, 182, and 230 of the study. ^a,b,c^indicates day (*P* < 0.05).

Treatment did not influence (*P* > 0.05) the total or pathogenic *E. coli* levels, which is similar to what was observed by Arthur et al., (2010) where no differences were observed between cattle that were either offered a *Bacillus*-based DFM or not offered a *Bacillus*-based DFM, as well as by Stefenoni et al. (2020) where there were no differences in *E. coli* prevalence between dairy cattle either offered YCW or not offered YCW. However, this contradicts Baines and Erb (2013) who found that beef feeder calves offered YCW reduced *E. coli* prevalence compared to calves not offered the YCW. There was a day effect where samples on days 1 and 14 had lower (*P* < 0.05) combined *E. coli* levels compared to the other sampling points but were not different (*P* > 0.05) from each other (Figure 3). Combined *E. coli* levels on day 77 were higher (*P* < 0.05) compared to days 133, 182, and 230. It has been suggested that seasonal differences in *E. coli* prevalence can be observed (Flach et al., 2022). As the first sampling day occurred in October, and the final sampling day was in June, this may explain the low levels recorded at the initial sampling time as *E. coli* prevalence is recorded to be greater during the summer months (Flach et al., 2022). It is important to note that pathogenic *E. coli* levels numerically decreased to similar or the lowest levels at the final sampling point (Table 2). Observing low levels is key, as it is unlikely to eliminate pathogens entirely; to at least reduce them to significantly lower levels would be considered an effective intervention (Arthur et al., 2007). Pathogenic *E. coli* levels of this study (49.6%) were lower (*P *< 0.05) compared to the other beef samples in the Church and Dwight research database (62.6%). Although there were no differences between control steers and steers receiving the DFM and/or YCW as reported in previous studies (Tabe et al., 2008; Flach et al., 2022), the levels of *E. coli* observed in the current study from all treatment groups were less compared to untreated controls (Tabe et al., 2008). Due to this low prevalence, it seems plausible as to why there were no differences observed in the current study.

### *Salmonella* prevalence

**Figure 4.**
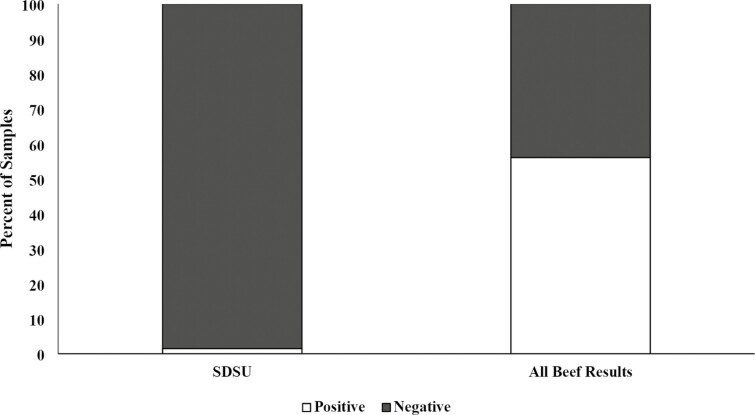
Percent of presumptive *Salmonella* prevalence in fecal samples collected from all treatments throughout a 258-d study compared with all of the beef results in the Church and Dwight research database. Treatments included: fed no DFM and no YCW (CON); fed only the DFM (DFM; Certillus CP B1801 Dry, *Bacillus subtilis* and *Lactobacillus plantarum* based DFM; 28 g/steer d^−1^ ); fed only the YCW (YCW; Celmanax, enzymatically hydrolyzed yeast component of *Saccharomyces cerevisiae*; 18 g/steer d^−1^ ); and fed the DFM and the YCW (DFM+YCW; Certillus 28 g/steer d^−1^ and Celmanax 18 g/steer d^−1^ of Celmanax). SDSU fecal samples had 1.5% *Salmonella* prevalence compared to 56.1% prevalence in the All Beef Results in the Church and Dwight research database.

There were no treatment × day interactions or treatment or day effects (*P* > 0.05) observed as there was very low *Salmonella* prevalence throughout the trial (1.5%). This is similar to results observed by Stefenoni et al. (2020) where no *Salmonella* prevalence difference was observed between treatment groups, and little *Salmonella* prevalence was observed throughout the study. *Salmonella* prevalence from all samples throughout the trial was 1.5%, compared to the Church and Dwight research database (56.1%; Figure 4). However, it is important to note that the *Salmonella* prevalence in the Church and Dwight research database is biased due to repetitive sampling at *Salmonella*-challenged sites. The little *Salmonella* observed in the present study is consistent with other data in the Northern Plains (Smith et al., 2021). The only time *Salmonella* was detected by Smith et al. (2021) was when there was a heat event observed during fecal sampling. The lack of *Salmonella* detected in the present study could be attributed to sampling that did not occur during a heat event. Steers were also directly shipped to the research facility from the source of origin and were not processed through an auction facility. Thus, this resulted in no exposure to pathogens from other cattle, as was observed with the cattle that came through an auction facility in the study by Smith et al. (2021). Still, *Salmonella* has been suggested to be random with its prevalence and shedding (Tabe et al., 2008), and warrants further investigation.

## Conclusion

Collectively, there were no appreciable treatment influences on the analyzed bacterial populations and the use of DFM and YCW had minimal effects on overall microbial prevalence. These data further indicate a low pathogenic bacterial challenge at the trial site, which could partially explain the lack of differences with DFM or YCW supplementation. Overall, the effects of YCW and DFM used alone or in combination cannot be expected to show additional benefits when animals are relatively unstressed with low pathogen loads.

## Data Availability

Data can be made available upon reasonable request to Z.K.S.

## Abbreviations

DFM: direct-fed microbial
PCR: polymerase chain reaction
RNC: Ruminant Nutrition Center
TSB: tryptic soy broth
YCW: yeast cell wall
